# Combined treatment of spinal cord injury using channeled Porous-GelMA scaffold loaded with genetically engineered MSCs expressing inducible ChABC and constitutive BDNF

**DOI:** 10.3389/fbioe.2026.1838918

**Published:** 2026-08-10

**Authors:** Zhongqing Ji, Jinming Liu, Jianwei Xu, Yu Zhang, Wentao Zhong, Ya’nan Hu, Huanxiang Zhang, Yixin Shen

**Affiliations:** 1 Department of Orthopedic Surgery, Second Affiliated Hospital of Soochow University, Suzhou, Jiangsu, China; 2 Department of Orthopedic Surgery, Suzhou Yongding Hospital, Suzhou, Jiangsu, China; 3 Department of Cell Biology, MOE Key Laboratory of Geriatric Diseases and Immunology, Suzhou Medical College of Soochow University, Suzhou, China; 4 Guizhou Medical University, Guiyang, China; 5 Jiangsu Key Laboratory of Organoid Engineering and Precision Medicine, Division of Nanobiomedicine, Chinese Academy of Sciences Suzhou Institute of Nano-tech and Nano-Bionics, Suzhou, China

**Keywords:** BDNF, ChABC, channeled porous-GelMA scaffold, mesenchymal stem cells, spinal cord injury

## Abstract

**Objective:**

Following spinal cord injury (SCI), glial scarring mediated by chondroitin sulfate proteoglycans (CSPGs), coupled with insufficient neurotrophic support, hinders axonal regeneration. Furthermore, the lack of physical support at the injury site during the acute phase compromises cell survival and engraftment. To overcome these challenges, we developed a three-dimensional channeled porous-GelMA scaffold (CPGS) that provides spatial guidance and structural support. This scaffold is integrated with genetically engineered MSCs designed to continuously release brain-derived neurotrophic factor (BDNF) and inducibly express Chondroitinase ABC (ChABC). This integrated system combines therapeutic factors, stem cells, and tissue engineering to improve the injured microenvironment and promote neural regeneration after SCI.

**Methods:**

MSCs were genetically engineered for continuous BDNF expression and doxycycline (Dox)-inducible ChABC expression. RT-qPCR and Western blotting were used to validate expression levels. We performed transcriptomic analysis and protein-level verification to assess the activation of key signaling pathways. A porous GelMA-based CPGS, featuring longitudinal guidance cues, was fabricated and characterized for its structure and mechanical properties. BDNF/ChABC-MSCs were then seeded into the CPGS to create an integrated system, which was subsequently evaluated in a rat SCI model.

**Results:**

The BDNF/ChABC-MSCs successfully expressed BDNF and Dox-inducible ChABC, leading to the activation of mTOR, Hedgehog, FAK, and MAPK signaling pathways, as demonstrated by transcriptomic profiling and confirmed at the protein level. The CPGS exhibited longitudinal guidance and favorable mechanical properties. *In vivo* studies showed that the BDNF/ChABC-MSCs–CPGS system significantly improved BBB scores, markedly reduced lesion size and collagen deposition, decreased CS-56 expression, and increased expression of the axonal regeneration markers, NF-H and GAP43.

**Conclusion:**

The BDNF/ChABC-MSCs–CPGS integrated system enhances the SCI lesion microenvironment through structural guidance and biochemical modulation, thereby promoting axonal regeneration and functional recovery. This approach presents a novel strategy for combined tissue engineering and stem cell therapy in SCI treatment.

## Graphical Abstract

**Figure:**
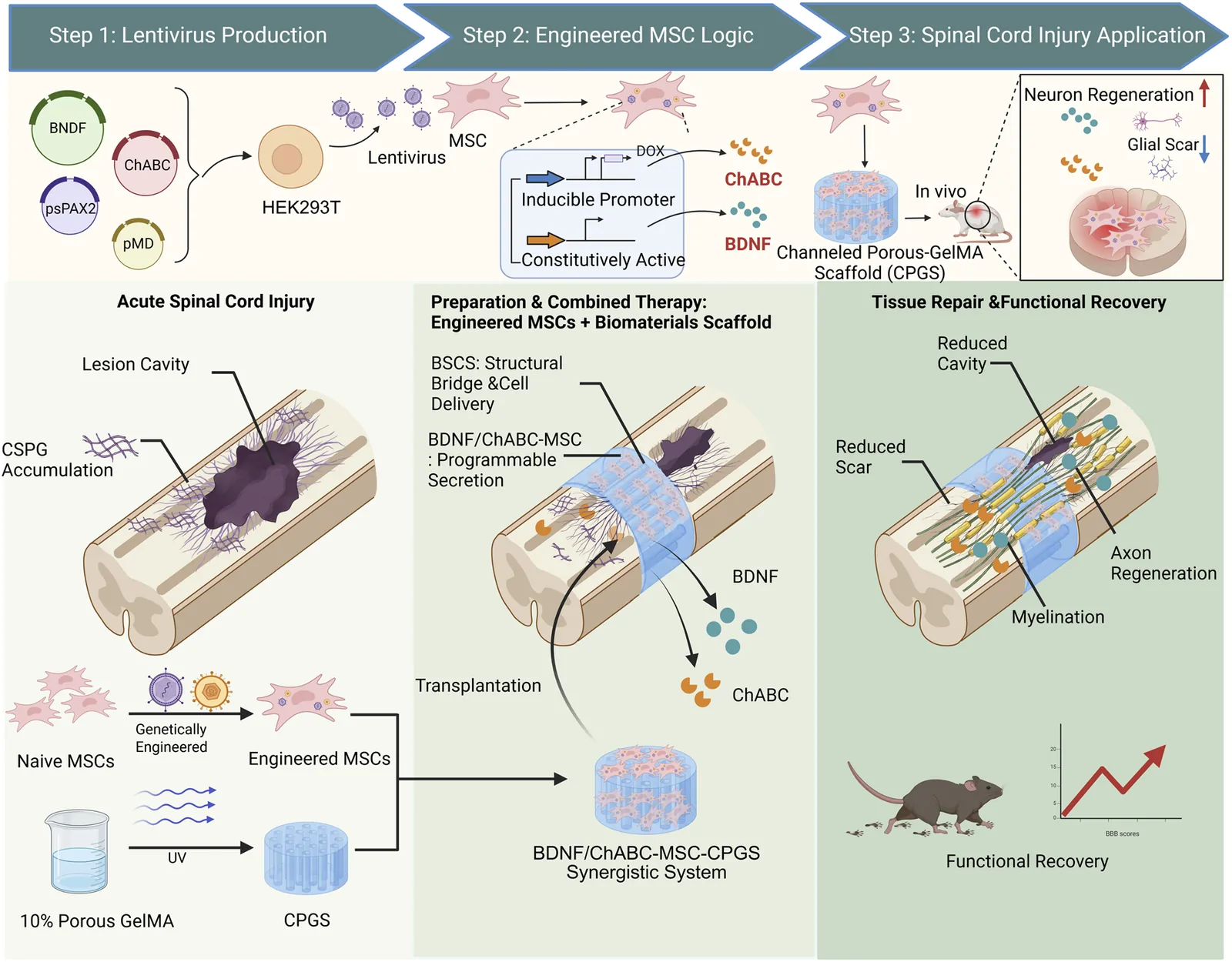
This study developed a combined therapeutic system to repair spinal cord injuries (SCI) by integrating genetically engineered MSCs into channeled porous-GelMA scaffolds (CPGS). The MSCs were programmed via lentiviral transduction to secrete ChABC under the control of Doxycycline induction and to constitutively express BDNF. After transplantation into rats with acute SCI, this CPGS-based system effectively remodeled the inhibitory microenvironment by reducing CSPG-rich scars and lesion cavities, which promoted axon regeneration and significant functional recovery.

## 1 Introduction

Spinal cord injury (SCI) often leads to lasting motor and sensory impairments below the site of injury, posing a significant challenge for neuroregenerative therapies (Wu et al., 2025). After SCI, regeneration is severely limited by the inhibitory microenvironment at the lesion site, characterized by structural barriers combined with biochemical suppressors (Hu et al., 2023). Reactive astrocytes secrete chondroitin sulfate proteoglycans (CSPGs), depositing them into the lesion’s extracellular matrix (ECM). There, CSPGs become a key component of the glial scar, forming a physical and molecular barrier that inhibits axonal extension (Cieri et al., 2025). Insufficient neurotrophic support in the injured spinal cord also limits neuronal viability and the restoration of synaptic connectivity (Haratizadeh et al., 2025). Consequently, mitigating inhibitory matrix deposition following scar formation and ensuring adequate trophic support are widely considered pivotal strategies for SCI repair (Chen et al., 2024a; Covache-Busuioc et al., 2025; Clifford et al., 2023).

Mesenchymal stem cells (MSCs) have been investigated as cellular carriers for the delivery of therapeutic factors in SCI (Xia et al., 2023). Chondroitinase ABC (ChABC) breaks down glycosaminoglycan chains in CSPGs, reducing inhibitory effects from the extracellular matrix. Brain-derived neurotrophic factor (BDNF) promotes neuronal survival, axonal growth, and synaptic remodeling, creating a more favorable environment for regeneration (Sharifi et al., 2022; Takiguchi et al., 2022; Deznabi et al., 2023). In this study, we genetically engineered MSCs to secrete ChABC in a doxycycline-inducible manner and to provide sustained release of BDNF.

This strategy harnesses genetic regulation to optimize the temporal profile of factor exposure, preserving the immunomodulatory and paracrine properties of MSCs. This approach mitigates the limitations of conventional local administration, such as a short half-life and unstable local concentrations (Trigo et al., 2025; Varkouhi et al., 2020). Nevertheless, MSC transplantation alone is limited in SCI lesions due to restricted cell survival, suboptimal engraftment, and insufficient structural guidance. Tissue-engineered scaffolds offer mechanical support and a defined three-dimensional architecture for cell-based therapies. This improves cell retention and transplantation efficiency, while also providing physical guidance cues for regenerating axons (Chen et al., 2024a; Sousa et al., 2023). Based on these considerations, we developed a three-dimensional, longitudinally channeled hydrogel scaffold using porous GelMA. Channelized and anisotropic scaffolds have been widely explored for SCI repair because aligned channels, fibers, or pores can provide topographical guidance for axonal extension. However, existing fabrication strategies still face practical limitations, including insufficient mechanical stability of soft hydrogel constructs, process-dependent constraints in additive manufacturing, and variability in fiber diameter, porosity, and alignment in electrospun conduits (Yuan et al., 2022; Behtaj et al., 2022). Therefore, we adopted a thermal-assisted micro-templated casting strategy to generate a monolithic longitudinally channeled GelMA scaffold with preserved channel patency and matrix integrity. This material vehicle was subsequently integrated with genetically engineered BDNF/ChABC-MSCs. This composite system combines the structural guidance provided by the CPGS with the spatiotemporally controlled delivery of factors by MSCs to investigate its proof-of-concept potential in modulating the lesion microenvironment, with the limitation that the current design cannot determine the independent or interactive contribution of each individual component.

## 2 Methods

### 2.1 MSC isolation, culture, characterization, and neuronal induction

MSCs were isolated from 6-week-old Sprague–Dawley rats (100–150 g) obtained from the Experimental Animal Center of Soochow University. All animal procedures were performed in accordance with institutional guidelines and approved by the Veterinary Administration of Soochow University. MSC isolation, expansion, and phenotypic identification were performed according to previously published protocols (Hu et al., 2024a). To ensure cellular stability and reproducibility, all experiments were performed using cells from passages 3 to 10. Immunophenotypic analysis of the isolated MSCs confirmed expression of the mesenchymal markers CD105 and CD29, and minimal expression of the hematopoietic markers CD34 and CD45 (Xu et al., 2022; Yusop et al., 2018).

For neuronal induction, MSCs were seeded at an initial density of 1 × 10^5^ cells/mL in low-glucose DMEM supplemented with 10% fetal bovine serum (FBS). After 24 h, the culture medium was replaced with a defined neuronal induction medium based on the N3 formulation. This medium consisted of DMEM/F12 supplemented with insulin (25 μg/mL), transferrin (50 μg/mL), sodium selenite (30 nM), progesterone (20 nM), putrescine (100 nM), basic fibroblast growth factor (bFGF; 10 ng/mL), valproic acid (0.5 mM), vitamin C (50 μM), and forskolin (50 μM), as previously described. To further promote neuronal lineage commitment, the induction medium was supplemented with a small-molecule cocktail containing CHIR99021 (3 μM), Y27632 (5 μM), SB431542 (2 μM), and purmorphamine (1 μM). The induction medium was refreshed every 2 days throughout the differentiation process (Liu et al., 2024).

### 2.2 Lentiviral preparation and transduction

Lentiviral vectors were generated in HEK293T cells via polyethylenimine (PEI)-mediated transient transfection. In brief, HEK293T cells at approximately 80% confluence were co-transfected with transfer plasmids encoding CMV-Tet3G, CMV-Tet3G-BDNF, Tre3GV/TetO-GFP, or Tre3GV/TetO-ChABC-Flag-GFP, and packaging plasmids psPAX2 and pMD2. G at predetermined ratios. Viral supernatants were harvested and subsequently concentrated by ultracentrifugation to obtain high-titer lentiviral preparations. For lentiviral transduction, target cells at approximately 70% confluence were exposed to concentrated viral particles at a multiplicity of infection (MOI) of 10 in the presence of polybrene (4 μg/mL). Twenty-four hours post-infection, the culture medium was replaced with fresh growth medium, and cells were passaged 48 h post-transduction. For experiments requiring inducible gene expression, Doxycycline (Dox; 500 ng/mL; Sigma) was added to the culture medium as indicated to activate ChABC expression and secretion.

### 2.3 RNA extraction, RT-qPCR, and RNA-Sequencing analysis

Total RNA was isolated using TRIzol reagent (Invitrogen) according to the manufacturer’s instructions. RNA concentration and purity were assessed using a NanoDrop microvolume UV spectrophotometer to ensure suitability for downstream applications. To perform reverse transcription quantitative PCR (RT-qPCR) analysis, equal amounts of total RNA were reverse-transcribed into complementary DNA (cDNA) using a commercial reverse transcription kit (Thermo Fisher Scientific) according to the manufacturer’s protocol. Quantitative PCR was subsequently performed using SYBR® Premix Ex Taq™ (Takara). Gene expression levels were normalized to GAPDH as an internal reference, and relative transcript abundance was calculated using the 2^^−ΔΔCt^ method. Primer sequences used for RT-qPCR are provided in Table 1.

**TABLE 1:** Primer sequences used for RT-qPCR analysis.

Genes	Sense	Anti-sense
*BDNF*	TTC​TGC​TGG​AGG​AAT​ACA​A	ACT​CGC​TAA​TAC​TGT​CAC​A
*ChABC*	AGA​GCC​GTA​GGC​GTC​TCT​CT	AGC​GTT​GAG​GGT​CAT​CTC​TC
*Tet3G*	TCAATGGAGTCGGTATCG	GCAGGAGTGGGTATGATG

To gain a comprehensive understanding of transcriptional changes, we performed RNA sequencing (RNA-seq) analysis. Total RNA was extracted as described above, and eukaryotic mRNA was enriched using Oligo (dT) magnetic beads. The purified mRNA was then fragmented into short fragments and reverse-transcribed into cDNA using the NEBNext Ultra RNA Library Prep Kit for Illumina (New England Biolabs). Following second-strand synthesis, the resulting double-stranded cDNA fragments underwent end-repair, A-tailing, and ligation to Illumina sequencing adapters. After purification with AMPure XP beads, the libraries were amplified by PCR and sequenced on an Illumina NovaSeq 6,000 platform by Gene *Denovo* Biotechnology Co. (Guangzhou, China).

RNA-seq data were analyzed with GSEA (v2.2.4). PCA was performed to visualize transcriptional profiles among GFP control, BDNF-MSCs, ChABC-MSCs, and BDNF/ChABC-MSCs, with biological replicates per group. To fundamentally avoid prospective inter-batch variability, all RNA-sequencing samples across the experimental groups were processed, library-prepared, and sequenced simultaneously within a single, unified batch. Raw sequencing read counts were normalized using the median-of-ratios method within the DESeq2 package to systematically account for variations in sequencing depth and gene length prior to downstream expression profiling. DEGs were identified using FDR-adjusted criteria (q < 0.05), followed by GO and KEGG pathway enrichment analyses using DAVID. For Gene Set Enrichment Analysis (GSEA), an exploratory loose threshold of q < 0.25 was intentionally utilized strictly as a preliminary screening mechanism for large-scale transcriptomic data. This high-throughput pre-screening was integrated into our experimental design solely to narrow down the target scope and discover candidate pathways for subsequent empirical testing; it was not employed as a metric for standard statistical significance, and all derived candidates mandated independent translation-level validation. The transcriptome data generated in this study have been deposited in the NCBI database under BioProject accession number PRJNA1430096.

### 2.4 Western blot analysis

Cells were rinsed with phosphate-buffered saline (PBS) and then lysed on ice using lysis buffer supplemented with phenylmethylsulfonyl fluoride (PMSF). Cellular debris was removed by centrifugation at 12,000 × g for 15 min at 4 °C. To ensure equal protein loading, the total protein concentration of the resulting supernatants was determined using a BCA protein assay kit (Thermo Fisher Scientific, USA), following the manufacturer’s instructions. Protein samples were then mixed with reducing sample buffer containing β-mercaptoethanol and denatured by heating at 95 °C for 10 min. Equal amounts of protein were separated by SDS–polyacrylamide gel electrophoresis and transferred to polyvinylidene fluoride (PVDF) membranes.

After transfer, membranes were blocked for 1 h at room temperature using 5% non-fat milk in Tris-buffered saline with 0.1% Tween-20 (TBST). Subsequently, membranes were incubated overnight at 4 °C with primary antibodies against: BDNF (Abcam, ab108319; 1:1,000), DDDDK tag (Flag) (Abcam, ab205606; 1:1,000), p-mTOR (Proteintech, 67778; 1:1,000), mTOR (Proteintech, 66888; 1:1,000), GLI1 (Proteintech, 66905; 1:1,000), p-FAK (Tyr397) (Cell Signaling Technology, 8,556; 1:1,000), FAK (Total) (Cell Signaling Technology, 3,285; 1:1,000), p-JNK (T183/Y185) (Cell Signaling Technology, 4,668; 1:1,000), JNK (Total) (Cell Signaling Technology, 9,252; 1:1,000), p-ERK1/2 (T202/Y204) (Cell Signaling Technology, 4,370; 1:1,000), ERK1/2 (Total) (Cell Signaling Technology, 9,102; 1:1,000), p-p38 (T180/Y182) (Cell Signaling Technology, 4,511; 1:1,000), p38 (Total) (Cell Signaling Technology, 8,690; 1:1,000), and β-actin (Proteintech, 66009-1-Ig; 1:20,000). Following the washing step, membranes were incubated for 1 h at room temperature with horseradish peroxidase-conjugated secondary antibodies. Immunoreactive bands were then visualized using enhanced chemiluminescence (ECL) reagents and captured with a chemiluminescence imaging system.

### 2.5 Fabrication of the channeled porous-GelMA scaffold (CPGS)

Lyophilized porous methacrylated gelatin (GelMA; EFL, Suzhou, China) was dissolved in PBS to obtain a 10% (w/v) solution.

To facilitate dissolution, the mixture was heated to 60 °C for 5 min, followed by vortex mixing for 5 min. This heating-mixing cycle was repeated three times to ensure complete homogenization. After homogenization, the GelMA solution was maintained at 60 °C until use. Cylindrical molds for scaffold fabrication were prepared in advance using 3D printing. The GelMA solution was then cast into the molds and exposed to ultraviolet light (405 nm, 3 W) for 45 s to induce photocrosslinking. To create aligned microchannels within the scaffold, nineteen filaments (Ø0.2 mm) were pre-positioned in the mold before crosslinking. After light curing, the sacrificial steel filaments were carefully extracted along the longitudinal axis, yielding a longitudinal guidance scaffold containing 19 aligned microchannels. The resulting channeled constructs were precisely sectioned into 2.0 mm functional lengths and thoroughly washed with PBS three times to remove any residual unreacted monomers before subsequent characterization and animal studies.

### 2.6 Scanning electron microscopy (SEM) analysis

For microstructural evaluation, cell-laden CPGS samples were fixed in 2% glutaraldehyde for 30 min at room temperature and then rinsed twice with PBS. The samples were then dehydrated using a series of graded ethanol solutions (30%, 50%, 70%, 80%, 90%, and 3 changes of 100%) for 10 min at each step, followed by two 5-min immersions in tert-butanol. After freezing in tert-butanol, the scaffolds were freeze-dried, sectioned longitudinally along the microchannel axis, and sputter-coated with gold. Microstructure was examined using a scanning electron microscope (S-4800, Hitachi, Japan) at an accelerating voltage of 3 kV.

### 2.7 Mechanical testing

Cylindrical specimens (Ø10 mm, 5 mm height) of 5% and 10% (w/v) GelMA, as well as porous GelMA formulations, were prepared to evaluate the compressive properties of GelMA-based hydrogels. After fabrication, samples were equilibrated in PBS at 37 °C for 24 h. Uniaxial compression tests were then performed using a universal testing machine (Mester Industrial Systems, China) at a loading rate of 0.2 min^-1^. Stress–strain curves were generated from force-displacement data using Origin software, and compressive moduli were calculated from the linear region of the curves between 5% and 10% strain. While uniaxial compression provides standard benchmarking data for hydrogel resistance to microchannel collapse, it does not evaluate tensile, shear, or viscoelastic behavior under dynamic spinal movement.

### 2.8 *In vitro* degradation and water retention analyses

To assess the *in vitro* degradation behavior of CPGS scaffolds, samples were immersed in PBS and incubated at 37 °C for up to 6 weeks, with the PBS solution replaced daily. At predetermined weekly intervals, scaffolds were collected, lyophilized, and weighed to determine the residual dry mass. The degradation rate (DR) was calculated as the percentage of mass loss relative to the initial dry weight using the following formula:

$$DR=\frac{W_{0}-W_{t}}{W_{0}}\times 100\%$$

where $W_{0}$ and $W_{t}$ represent the initial dry weight and the dry weight of the sample at a specific time point, respectively.

The water retention capacity was evaluated by initially measuring the dry weight of air-dried scaffolds (W_0_). Subsequently, the scaffolds were immersed in PBS at 37 °C for 24 h to achieve full saturation (W_1_). Following immersion, samples were removed from the PBS and maintained at 25 °C and 40% relative humidity, with weights recorded at predetermined intervals (W_t_).

Water retention (WR) was expressed as the percentage of retained water relative to the fully hydrated state and was calculated using the following equation:

$$WR=\frac{W_{t}-W_{0}}{W_{1}-W_{0}}\times 100\%$$

where $W_{0}$ and $W_{t}$ represent the initial dry weight and the weight of the sample at a specified time point after PBS immersion, respectively.

### 2.9 Cell attachment and morphological characterization

To evaluate the cytocompatibility of the CPGS, we used either BDNF/ChABC-MSCs pre-induced with doxycycline for 3 days or neural induction MSCs. The latter were pre-infected with a GFP lentivirus to express GFP. Before cell seeding, pre-fabricated CPGS were gently blotted with sterile filter paper to remove residual liquid. Cells were harvested and resuspended at a density of 2 × 10^6^ cells/mL. Then, 20 μL of the cell suspension was evenly seeded onto one surface of the scaffolds. After a 30-min incubation to allow initial cell attachment, the scaffold was inverted, and another 20 μL of cell suspension was applied to the opposite surface, followed by another 30-min incubation. Complete culture medium was then added to fully immerse the scaffold, and samples were maintained under standard culture conditions (37 °C, 5% CO_2_). Following 24 h of culture, cell-scaffold constructs were fixed in 4% paraformaldehyde, permeabilized with 0.2% Triton X-100, and blocked using 3% bovine serum albumin. Cytoskeletal organization and cell morphology within the CPGS were visualized by staining filamentous actin with phalloidin (1:2000, Invitrogen), followed by observation using confocal laser scanning microscopy (CLSM, Leica, Germany).

### 2.10 Spinal cord transection, scaffold implantation, and *in vivo* evaluation

Healthy, female Sprague-Dawley rats (180–200 g) were used for all *in vivo* experiments. The Institutional Animal Care and Use Committee approved all surgical and animal procedures, which were performed in accordance with established ethical guidelines. Animals were randomly assigned to one of four explicit experimental groups: 1. Sham Group (Blank Control): Animals underwent standard T9–T10 laminectomy to expose the spinal cord but did not receive any spinal cord injury, tissue resection, or scaffold/cell implantation. 2. SCI Group (Negative Control): Animals were subjected to a complete T10 spinal cord transection, creating a 2-mm physical tissue defect. The cavity was treated standardly for hemostasis using a gelatin sponge, without any materials implantation or cell transplantation. 3. CPGS Group (Scaffold-Only Control): Animals received the identical T10 transection (2-mm defect) followed by the immediate localized insertion of a cell-free CPGS to the lesion site. 4. BDNF/ChABC-MSCs–CPGS Group (Experimental Treatment Group): Animals received the T10 transection (2-mm defect) followed by the immediate transplantation of the integrated CPGS pre-seeded with 2 × 10^6^ cells/mL of BDNF/ChABC-MSCs. This design serves as an evaluation of the integrated system rather than a full factorial analysis of each single component.

Rats were anesthetized using either isoflurane inhalation (2%–3%) or an intraperitoneal injection of pentobarbital sodium (50 mg/kg). The surgical area was then shaved and disinfected. A laminectomy was performed at the T9-T10 vertebral level to fully expose the spinal cord. A complete transection of the spinal cord was performed at the T10 level using a microsurgical blade under an operating microscope. This was achieved by making two parallel incisions approximately 2 mm apart, followed by careful removal of the intervening spinal tissue with fine forceps to create a 2-mm gap. After hemostasis was achieved using a gelatin sponge, the prepared CPGS (either cell-free or loaded with BDNF/ChABC-MSCs) was carefully implanted into the lesion cavity, ensuring that the scaffold microchannels were aligned parallel to the longitudinal axis of the spinal cord. The muscle and skin layers were then sutured in anatomical order.

Postoperative care included intramuscular administration of penicillin (50,000 U/day) for 3 days to prevent infection. Manual bladder expression was performed twice daily until reflexive urination returned. To induce the expression of BDNF and ChABC in all experimental groups, Doxycycline (Dox, 10 mg/kg) was administered via oral gavage every other day, starting 1 week post-surgery. The Dox was dissolved in a 2% (w/v) sucrose solution and protected from light during administration.

Crucially, while this surgery serves as a proof-of-concept evaluation of structural integration, the longitudinal cell survival, dynamic retention, and chronological fate of the seeded MSCs *in vivo* were not quantified in the current animal study protocol.

### 2.11 Behavioral assessment

Hindlimb motor function was evaluated using the 21-point Basso–Beattie–Bresnahan (BBB) locomotor rating scale (Basso et al., 1995). Behavioral assessments were rigorously conducted by two well-trained independent observers who were fully blinded to the specific experimental group assignments of the rats to eliminate any prospective investigator bias. Scoring sessions were conducted at identical post-injury intervals at predetermined time points for up to 6 weeks post-injury. The final assigned BBB score for each individual animal at each designated timepoint represents the exact average of these two independent blind assessments, demonstrating high inter-rater reliability. Parameters, including joint movement, stepping ability, coordination, and overall locomotor performance, were systematically scored. Each experimental group comprised at least six animals per time point.

### 2.12 Tissue processing, histological staining, and immunofluorescence analysis

Following completion of the experiments, animals were deeply anesthetized and transcardially perfused with 0.9% saline, followed by 4% paraformaldehyde (PFA) in PBS. Spinal cord segments containing the injury site were the harvested, fixed in 4% paraformaldehyde for 48 h, and cryoprotected in 30% (w/v) sucrose. Next, approximately 2-cm-long spinal cord segments, with the lesion epicenter at the midpoint, were dissected, carefully stripped of dura mater, embedded in OCT compound, and longitudinally sectioned at 25 μm thickness using a cryostat (Leica CM 1900). The sections were then mounted onto gelatin-coated slides for subsequent analyses.

For histological evaluation, hematoxylin and eosin (H&E) and Masson’s trichrome staining were performed according to established protocols (Zhang et al., 2026). For immunofluorescence analysis, tissue sections were blocked for 1 h at room temperature in PBS containing 5% bovine serum albumin and 0.3% Triton X-100. The sections were then incubated with primary antibodies against NF-H (Cell Signaling Technology, 2,836; 1:200), GAP43 (Cell Signaling Technology, 8,945; 1:200), and CS-56 (Invitrogen, MA1-83055; 1:200). Nuclei were counterstained with Hoechst 33258 (1:1,000; Sigma-Aldrich). Fluorescent images were acquired using a confocal microscope, and quantitative analysis of staining areas within defined regions of interest was performed using NIH ImageJ software (version 1.51).

### 2.13 Statistical analysis

Quantitative data are presented as mean ± standard error of the mean (SEM). Two-tailed Student's t tests were used to compare two groups. For multiple group comparisons, parametric data were analyzed via analysis of variance (ANOVA) followed by Bonferroni–Dunn *post hoc* tests (GraphPad Prism 9). Due to the ordinal nature of the BBB locomotor scale, behavioral scores were analyzed using non-parametric statistical methods to account for prospective floor and ceiling effects. Statistical significance was defined as *p* < 0.05 and *p* < 0.01, and significance levels are indicated as, # and*, ##, respectively. Each experiment was independently repeated at least three times unless otherwise noted.

## 3 Results

### 3.1 Construction of genetically engineered BDNF/ChABC-MSCs

**FIGURE 1:**
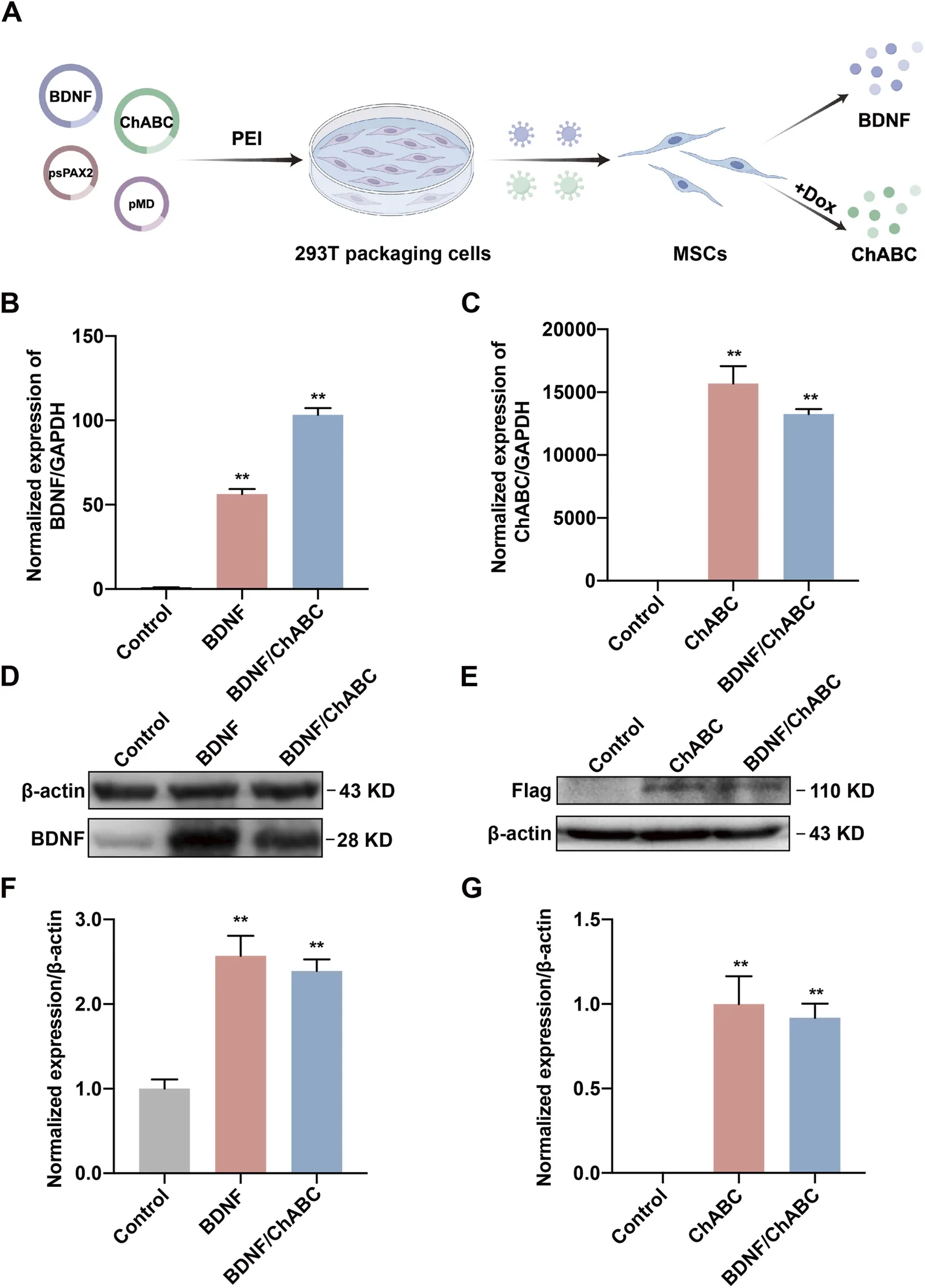
Construction and validation of genetically engineered BDNF/ChABC-MSCs with constitutive BDNF expression and Dox-inducible ChABC expression. **(A)** Schematic illustration of lentiviral vector production in HEK293T packaging cells and subsequent transduction of MSCs to generate engineered cells capable of sustained BDNF secretion and Dox-inducible ChABC expression. **(B)** RT-qPCR analysis of BDNF mRNA expression normalized to GAPDH in Control, BDNF, and BDNF/ChABC groups. **(C)** RT-qPCR analysis of ChABC mRNA expression normalized to GAPDH in Control, ChABC, and BDNF/ChABC groups. **(D)** Representative Western blot showing BDNF protein expression in the following groups: Control, BDNF, and BDNF/ChABC. β-actin served as the loading control. **(E)** Representative Western blot showing ChABC-Flag expression in the Control, ChABC, and BDNF/ChABC groups. β-Actin was used as a loading control. **(F)** Densitometric quantification of BDNF protein levels normalized to β-actin. **(G)** Densitometric quantification of ChABC-Flag protein levels normalized to β-actin. *P < 0.05, **P < 0.01 versus the control group.

To generate genetically engineered mesenchymal stem cells (BDNF/ChABC-MSCs) capable of sustained BDNF expression and Doxycycline (Dox)-inducible ChABC expression, we employed the Tet-On system. This system enables precise regulation of target gene expression through the addition of Dox (Das et al., 2016; Kalimuthu et al., 2017). We designed three key plasmids: CMV-Tet3G-BDNF for constitutive BDNF expression, Tre3GV/TetO-GFP as a reporter to monitor transfection efficiency, and Tre3GV/TetO-ChABC-Flag-GFP for Dox-induced expression of Flag-tagged ChABC. Therefore, in this system, BDNF is constitutively expressed, while ChABC expression is induced by Dox (Figure 1A).

RT-qPCR and Western blot analysis confirmed constitutive BDNF expression and Dox-inducible ChABC expression in BDNF/ChABC-MSCs. Specifically, RT-qPCR showed stable BDNF expression and a significant increase in ChABC expression upon Dox induction (Figures 1B,C). Western blot analysis further verified these findings, demonstrating constitutive expression of BDNF (28 kDa) and high expression of an approximately 110 kDa Flag-tagged ChABC in the Dox-treated group (Figures 1D,E). This detected molecular weight is consistent with the full-length bacterial ChABC derived from *Proteus* vulgaris, although molecular weight alone serves as a necessary proxy rather than definitive confirmation of structural folding or functional enzymatic kinetics. Quantitative analysis revealed significantly higher levels of both BDNF and ChABC compared to controls (Figures 1F,G). These results confirm the successful generation of MSCs capable of constitutive BDNF expression and Dox-inducible ChABC expression, providing a reliable cell model for subsequent *in vitro* and *in vivo* experiments.

### 3.2 Combined activation of mTOR, hedgehog, FAK, and MAPK signaling pathways in BDNF/chabc-mscs

**FIGURE 2:**
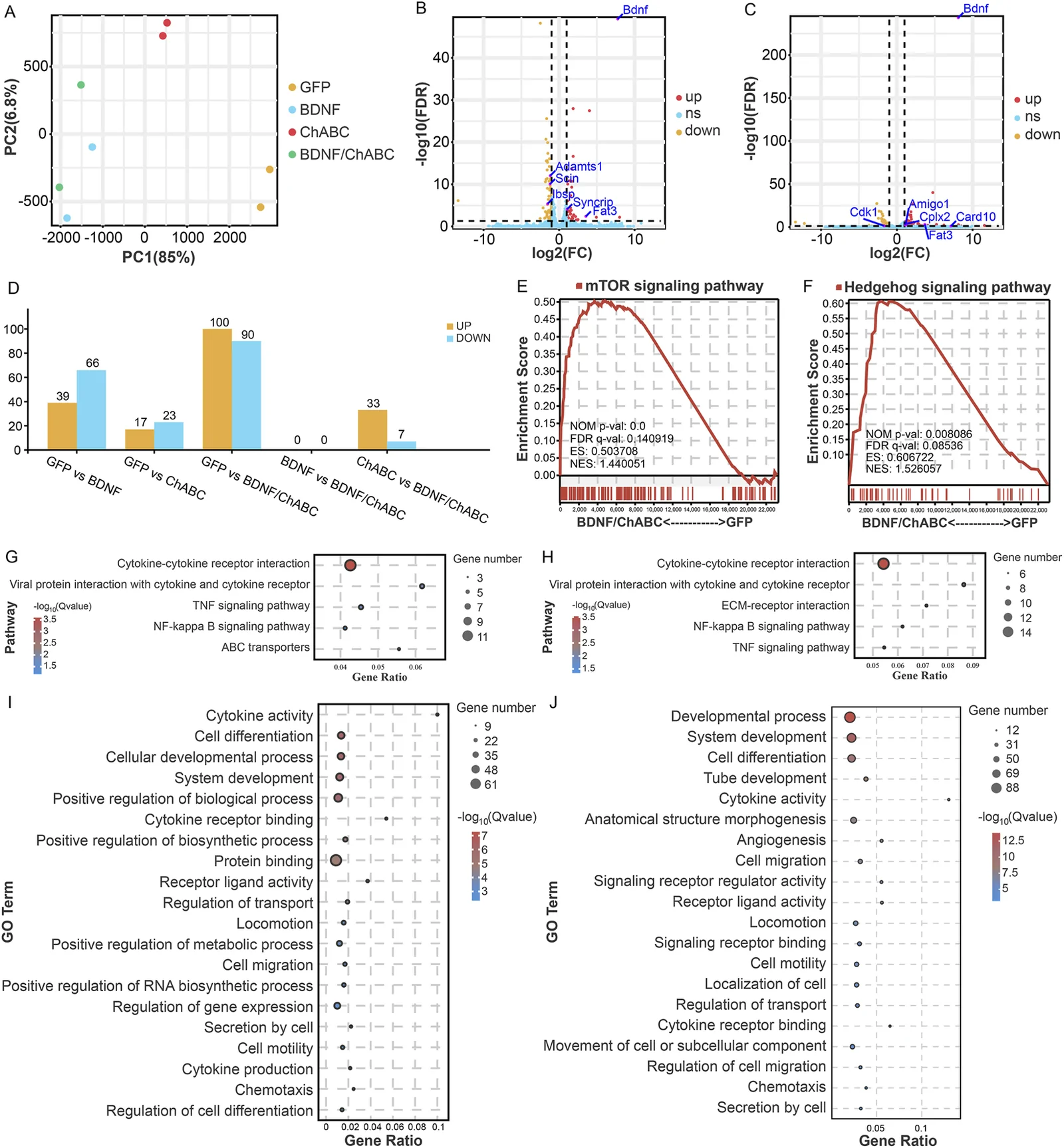
Transcriptomic profiling of engineered MSCs identifies pathway-level remodeling associated with BDNF/ChABC co-expression. **(A)** Principal component analysis (PCA) of RNA-seq data from GFP (Control), BDNF, ChABC, and BDNF/ChABC groups. **(B)** Volcano plot showing DEGs in BDNF vs GFP. **(C)** Volcano plot showing DEGs in BDNF/ChABC vs GFP. **(D)** A summary of the DEG counts (upregulated and downregulated genes) across pairwise comparisons (GFP vs BDNF, GFP vs ChABC, GFP vs BDNF/ChABC, BDNF vs BDNF/ChABC, and ChABC vs BDNF/ChABC). **(E)** Gene set enrichment analysis (GSEA) demonstrating the positive enrichment of the mTOR signaling pathway in BDNF/ChABC vs GFP. **(F)** GSEA showing positive enrichment of the Hedgehog signaling pathway in BDNF/ChABC vs GFP. **(G)** KEGG pathway enrichment analysis of DEGs in BDNF vs GFP. **(H)** KEGG pathway enrichment analysis of DEGs in BDNF/ChABC vs GFP. **(I)** Gene Ontology (GO) enrichment analysis of DEGs in BDNF vs GFP. **(J)** GO enrichment analysis of DEGs in BDNF/ChABC vs GFP.

To elucidate the effects of BDNF, ChABC, and their combined overexpression on MSC cellular function and the associated molecular mechanisms, we performed a comprehensive transcriptomic analysis of MSCs from each treatment group. PCA was used as an exploratory visualization method among GFP control, BDNF-MSCs, ChABC-MSCs, and BDNF/ChABC-MSCs. Although PC1 accounted for 85% of total variance, this does not establish biological significance. DEG analysis revealed a modest transcriptional response in BDNF/ChABC-MSCs compared with GFP-MSCs, suggesting effects may involve limited transcriptional changes, ECM regulation, and post-translational signaling (Figure 2A). Differential gene expression (DEG) analysis supported these findings, demonstrating a clear clustering of upregulated and downregulated transcripts in the BDNF/ChABC group (Figures 2B,C). Specifically, comparison of GFP vs. BDNF/ChABC revealed 100 upregulated and 90 downregulated genes, a greater number than GFP vs. BDNF (39 up/66 down) and GFP vs ChABC (17 up/23 down). No significant DEGs were observed between BDNF-MSCs and BDNF/ChABC-MSCs, indicating that the additional effects of ChABC in the BDNF background may primarily occur via extracellular matrix modulation or post-translational signaling rather than transcriptional changes. In contrast, the ChABC-MSCs versus BDNF/ChABC-MSCs comparison identified 33 upregulated and 7 downregulated genes (Figure 2D), highlighting modest transcriptional alterations. Gene Ontology (GO) enrichment analysis indicated that these differentially expressed genes are primarily involved in biological processes critical for neural repair, including cell migration, cell differentiation, and cytokine activity (Figures 2I,J). Interestingly, the biological processes enriched in the BDNF group differed slightly from those in the BDNF/ChABC group. KEGG pathway enrichment analysis indicated that the BDNF group primarily showed cytokine-related pathway enrichment, whereas the BDNF/ChABC group exhibited enrichment of ECM-receptor interaction pathways (Figures 2G,H). All enrichment results were evaluated using FDR-adjusted p-values, and modest enrichment was interpreted with caution. Moreover, Gene Set Enrichment Analysis (GSEA) was implemented to screen for repair-relevant cascade trends. Utilizing an exploratory pre-screening threshold (q < 0.25) to interrogate the large-scale transcriptomic data, the mTOR pathway (NES = 1.44, FDR q = 0.14) and the Hedgehog pathway (NES = 1.52, FDR q = 0.08) were identified as potential candidate pathways for downstream investigation (Figures 2E,F). Crucially, these exploratory enrichment trends did not reach formal statistical significance under standard stringent false discovery rate metrics (q < 0.05). Consequently, these GSEA findings are treated strictly as non-significant, hypothesis-generating indicators that served to narrow our analytical scope, rather than transcriptomic proof of pathway activation. Definitive conclusions regarding functional pathway regulation were anchored strictly upon our subsequent standalone protein-level Western blot evaluations of key marker proteins, which provided the conclusive empirical validation of cascade activation.

**FIGURE 3:**
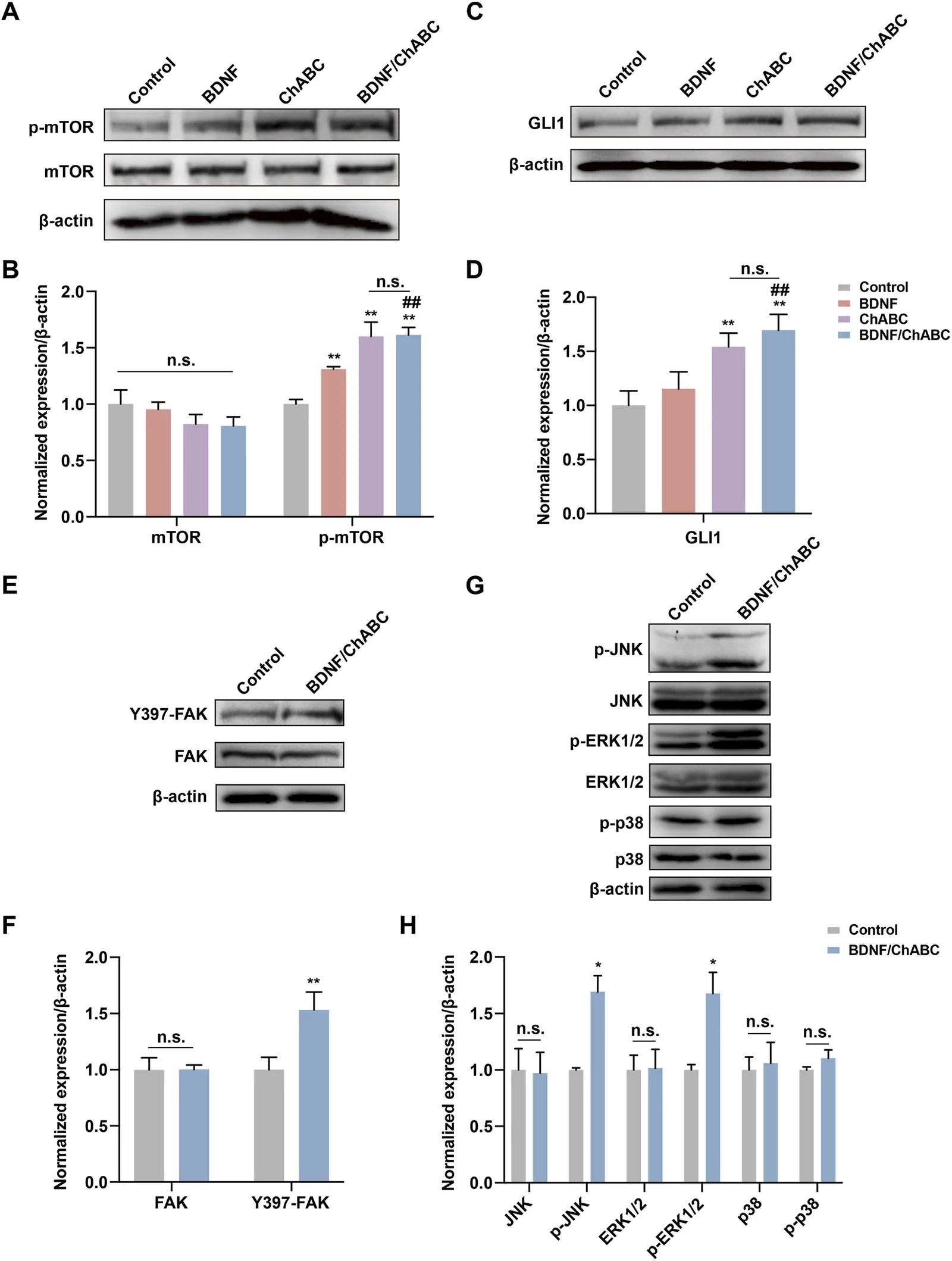
Protein-level validation of signaling pathway activation in BDNF/ChABC-MSCs. **(A)** Representative Western blots illustrating the total mTOR and phosphorylated mTOR (p-mTOR) in Control, BDNF, ChABC, and BDNF/ChABC groups, with β-actin as the loading control. **(B)** Densitometric quantification of total mTOR and p-mTOR normalized to β-actin. **(C)** Representative Western blot displaying the GLI1 expression in Control, BDNF, ChABC, and BDNF/ChABC groups, with β-actin as the loading control. **(D)** Densitometric quantification of GLI1 normalized to β-actin. **(E)** Representative Western blots indicating the total FAK and phosphorylated FAK (Y397-FAK) in Control and BDNF/ChABC groups, with β-actin as the loading control. **(F)** Densitometric analysis of the total FAK and Y397-FAK normalized to β-actin. **(G)** Representative Western blots illustrating MAPK pathway proteins (JNK, p-JNK, ERK1/2, p-ERK1/2, p38, and p-p38) in Control and BDNF/ChABC groups, with β-actin as the loading control. **(H)** Densitometric quantification of MAPK pathway proteins normalized to β-actin. *P < 0.05, **P < 0.01 versus the indicated control group; ##*P* < 0.01 versus the BDNF group; n. s., not significant.

To validate the activation of the mTOR and Hedgehog pathways suggested by our transcriptomic analysis, we performed Western blot analysis to assess the protein levels of key signaling pathway components. Consistent with the GSEA data, we found that GLI1, the terminal effector protein of the Hedgehog pathway, was significantly upregulated in the BDNF/ChABC group (Figures 3C,D). Additionally, we observed a significant increase in the phosphorylation level of mTOR (p-mTOR), while total mTOR protein levels remained stable, which further confirms the activation of the mTOR signaling pathway (Figures 3A,B).

Beyond the mTOR and Hedgehog pathways, we also investigated the focal adhesion kinase (FAK) and MAPK signaling pathways, which are associated with cell migration. Western blot analysis revealed a significant increase in the phosphorylation level of FAK at the Y397 activation site in BDNF/ChABC-MSCs (Figures 3E,F), aligning with the enrichment of cell motility-associated genes observed in the GO analysis. Protein-level assessment indicated that phosphorylation of JNK and ERK1/2 in the MAPK pathway was significantly increased in the BDNF/ChABC group (Figures 3G,H), while p-p38 levels remained unchanged, confirming the specific activation of the MAPK pathway at the protein level.

In conclusion, the concurrent increase in p-FAK, p-mTOR, p-JNK, p-ERK1/2, and GLI1 suggests that BDNF/ChABC engineering is associated with activation-related changes in multiple repair-relevant signaling pathways. However, these data do not establish a linear causal pathway or formal synergy.

## 4 Material characterization of CPGS

**FIGURE 4:**
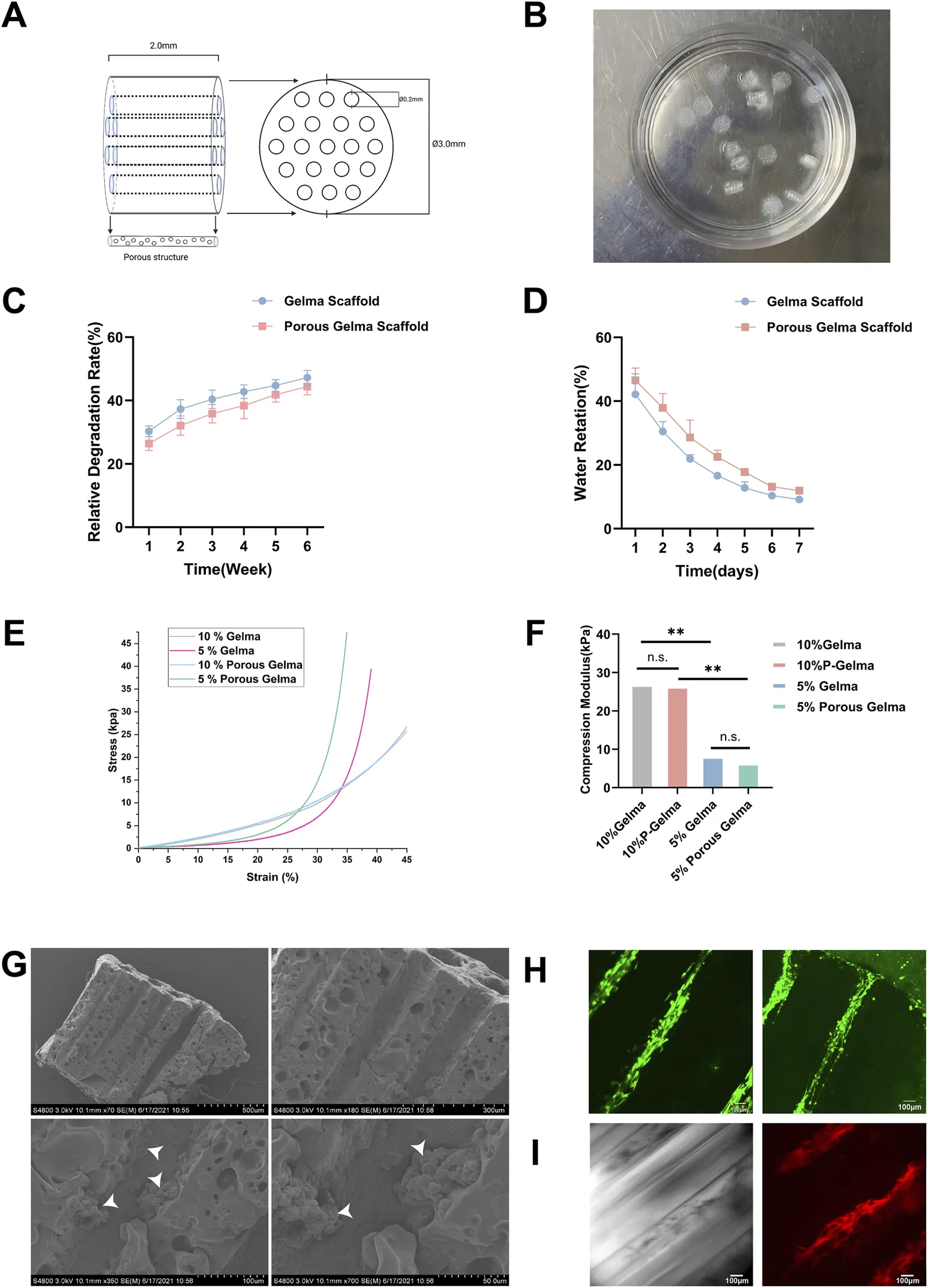
Design, material properties, and cytocompatibility of CPGS. **(A)** The CPGS architecture is schematically illustrated as a cylindrical scaffold (≈2.0 mm long and Ø3.0 mm) featuring 19 longitudinal through-channels (Ø0.2 mm) and a porous structure. **(B)** Representative photograph of the fabricated CPGS. **(C)** *In vitro* degradation profile of GelMA and porous GelMA scaffolds over 6 weeks, presented as relative degradation rate. **(D)** Water retention of GelMA and porous GelMA scaffolds measured over 7 days. **(E)** Representative compressive stress–strain curves of GelMA and porous GelMA scaffolds at different concentrations (5% and 10%). **(F)** Compressive modulus of GelMA and porous GelMA scaffolds at 5% and 10% concentrations. **(G)** Scanning electron microscopy images reveal the longitudinal microchannels and porous microstructure of the CPGS scaffold. Arrows indicate BDNF/ChABC-MSCs attached within the scaffold. **(H)** Representative fluorescence images showing GFP-positive BDNF/ChABC-MSCs (H1) and neural induction MSCs (H2) seeded on CPGS (scale bar = 100 μm). **(I)** Representative phase-contrast (I1) and F-actin staining (I2) images of MSCs on CPGS, illustrating cell adhesion and cytoskeletal organization (scale bar = 100 μm). **P < 0.01 versus the indicated control group.

To enhance the efficacy of BDNF/ChABC-MSC transplantation, channeled porous-GelMA scaffolds (CPGS) were engineered to offer structural support and a conducive microenvironment. The CPGS, depicted in Figure 4A, was designed as a cylindrical scaffold (approximately 2.0 mm long and Ø3.0 mm) containing 19 concentric, through-going longitudinal microchannels (Ø0.2 mm). This design established a straight, axially aligned guidance architecture. The fabricated CPGS exhibited a uniform appearance and consistent dimensions (Figure 4B), integrating longitudinal through-channels with an interconnected porous network (Figures 4A,G).

During *in vitro* stability testing, the CPGS displayed a gradual mass loss over 6 weeks, with a relative degradation rate similar to that of conventional GelMA scaffolds and without any significant difference (Figure 4C). This 6-week degradation window was intentionally selected to temporarily cover the critical subacute phase of matrix remodeling and early axonal sprouting in rat SCI, though long-term *in vivo* scaffold remodeling was not monitored. This suggests that the porous channelized architecture did not substantially alter the overall degradation behavior. Concurrently, the CPGS exhibited a relatively higher tendency for water retention during dehydration (Figure 4D), indicating its capacity to maintain hydration over a defined period. This property is crucial for providing a stable microenvironment that supports cell viability and solute diffusion. Mechanical characterization revealed that the GelMA concentration was the primary determinant of scaffold strength, with the 10% (w/v) Porous-GelMA and non-porous GelMA groups demonstrating significantly higher compressive moduli (mean values of approximately 26.4 kPa and 26.9 kPa, respectively) compared to the corresponding 5% (w/v) formulations, which exhibited values clustered between 5.2 kPa and 8.6 kPa (Figures 4E,F). While lower-stiffness matrices should better mimic native neural tissue, initial observations revealed that the 5% (w/v) formulations lacked the mechanical strength to prevent microchannel collapse under simulated physiological conditions. Furthermore, their poor surgical handling hindered their clinical applicability. The 10% (w/v) groups, however, exhibited greater structural integrity.

Notably, statistical analysis showed that the introduction of porosity (Porous-GelMA Group) did not significantly reduce the compressive modulus compared to non-porous formulations.

This confirms that 10% Porous-GelMA successfully maintains overall mechanical stability while providing a porous structure (Figure 4F). Consequently, 10% Porous-GelMA was selected for subsequent studies to ensure persistent physical support and prevent microchannel collapse under physiological conditions. Although this compressive modulus (averaging ∼26.4 kPa) remains higher than the reported local stiffness values for soft native mammalian spinal cord tissue substances (approximately 0.2–1.0 kPa) (Fiford and Bilston, 2005), this moderate structural stiffness is mechanically necessary to guarantee baseline surgical handleability and to maintain microchannel patency against host tissue compression.

SEM confirmed the presence of longitudinal through-channels accompanied by a uniform porous architecture, forming a continuous three-dimensional network across channel walls and interconnected pores. After seeding, BDNF/ChABC-MSCs adhered to the scaffold surface and within pore or channel regions, appearing as clustered cell aggregates (arrows) (Figure 4G), consistent with a microtopography conducive to cellular engraftment. Fluorescence imaging further showed that GFP-positive BDNF/ChABC-MSCs distributed along the longitudinal channels with an apparent alignment tendency (Figure 4H1). Importantly, neural-induced MSCs likewise maintained stable adhesion and distribution on the CPGS (Figure 4H2), indicating that the CPGS is biocompatible not only with BDNF/ChABC-MSCs but also likely with host neurons. Phase-contrast imaging, together with cytoskeletal staining, revealed cell spreading oriented along the channel axis with continuous F-actin organization (Figure 4I), collectively demonstrating that the CPGS provides a well-defined axial guidance microenvironment while supporting the adhesion and morphological maintenance of BDNF/ChABC-MSCs.

## 5 BDNF/ChABC-MSCs-CPGS promotes functional recovery and structural regeneration after rat spinal cord injury

**FIGURE 5:**
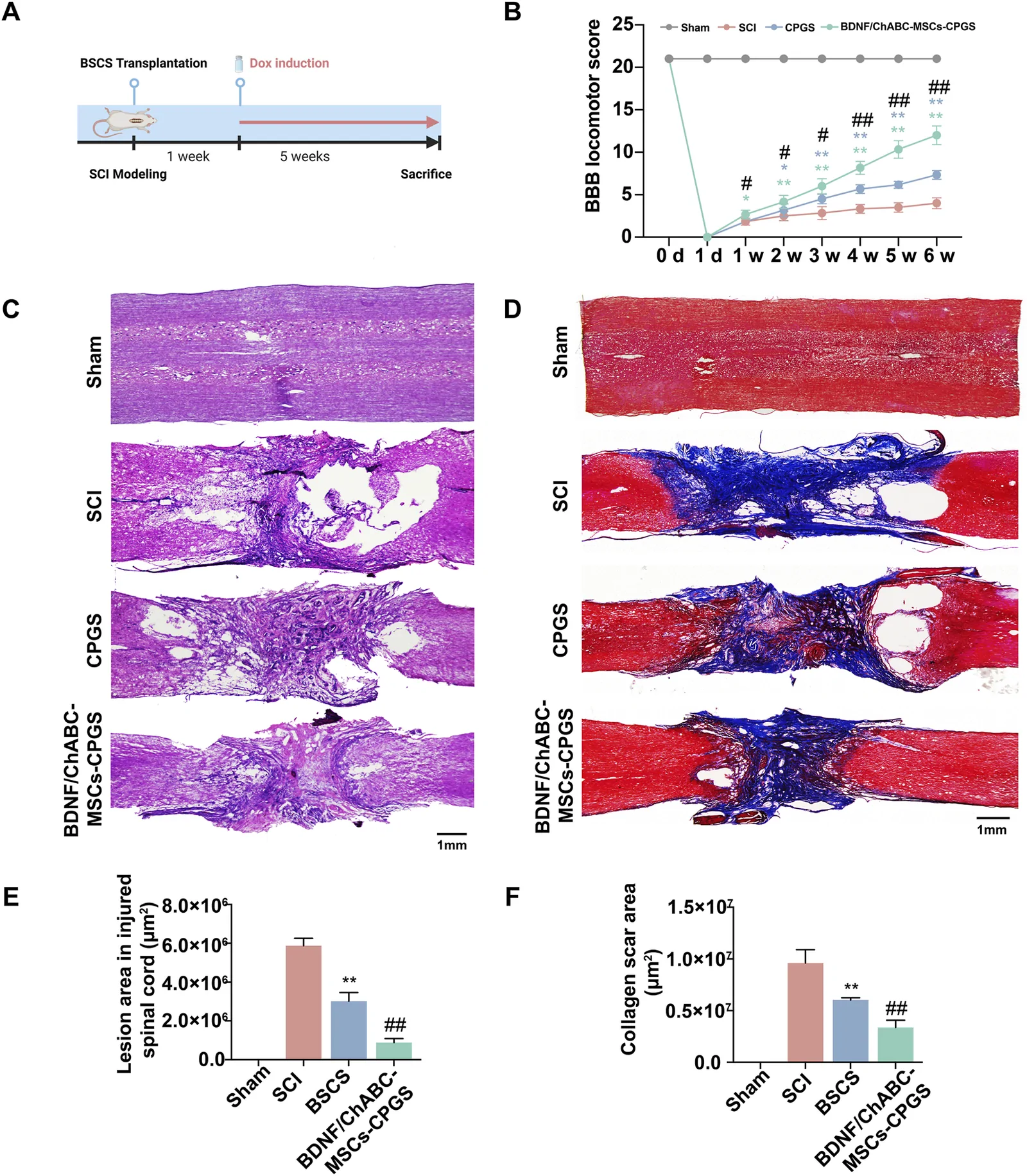
BDNF/ChABC-MSCs–CPGS improves locomotor recovery and attenuates lesion pathology after SCI. **(A)** Experimental timeline illustrating SCI induction, CPGS transplantation, initiation of Dox induction at 1week post-SCI to trigger ChABC expression. **(B)** BBB locomotor scores over time in Sham, SCI, CPGS, and BDNF/ChABC-MSCs–CPGS groups. **(C)** Representative H&E-stained longitudinal spinal cord sections illustrating tissue morphology and lesion cavity across groups. **(D)** Representative Masson’s trichrome-stained sections showing collagen deposition and scar formation across groups. **(E)** Quantification of lesion area in the injured spinal cord. **(F)** Quantification of collagen scar area. *P < 0.05, **P < 0.01 indicates a significant difference between the CPGS group and the SCI group; *P < 0.05, **P < shows a significant difference between the BDNF/ChABC-MSCs-CPGS group and the SCI group; #*P* < 0.05, ##*P* < 0.01 represents a significant difference between the BDNF/ChABC-MSCs-CPGS group and the CPGS group.

To perform a proof-of-concept evaluation of the integrated construct *in vivo*, we evaluated treatment outcomes using a rat T10 complete transection model. Following surgical ablation, the scaffold was carefully inserted into the 2-mm resection cavity as a structural bridge rather than being forcefully compressed into an intact spinal cord. The 2.0 mm length was selected to match this longitudinal defect, and the Ø 3.0 mm was designed to fit the exposed rat thoracic lesion cavity, with outcomes evaluated at an 8-week terminal post-mortem endpoint. One week post-injury, Dox induction was initiated via oral gavage every other day based on literature-derived dynamics of CSPG accumulation, though without direct experimental timing validation or pharmacodynamic tracking in this study (Figure 5A). We then monitored locomotor recovery over time and validated post-injury morphological and molecular phenotypes through histological staining and immunofluorescence. Behavioral assessments showed a gradual improvement in BBB scores across all groups.

BBB scores indicated improvement in gross locomotor performance in the BDNF/ChABC-MSCs-CPGS group compared with SCI and scaffold-only controls (Figure 5B). Interpretation is limited to gross motor recovery, without assessing fine motor coordination, proprioception, or electrophysiology.

These behavioral results were corroborated by histological analyses. Hematoxylin and eosin (H&E) staining showed that the BDNF/ChABC-MSCs-CPGS group had a smaller tissue defect and cavity area, as well as a more continuous lesion boundary, compared to controls (Figure 5C). Quantitative analysis confirmed a decrease in lesion area (Figure 5E). Furthermore, Masson’s trichrome staining revealed decreased collagen deposition and a reduced fibrotic scar burden in the BDNF/ChABC-MSCs-CPGS group (Figures 5D,F), suggesting that the composite system partially attenuates scarring and promotes tissue remodeling.

**FIGURE 6:**
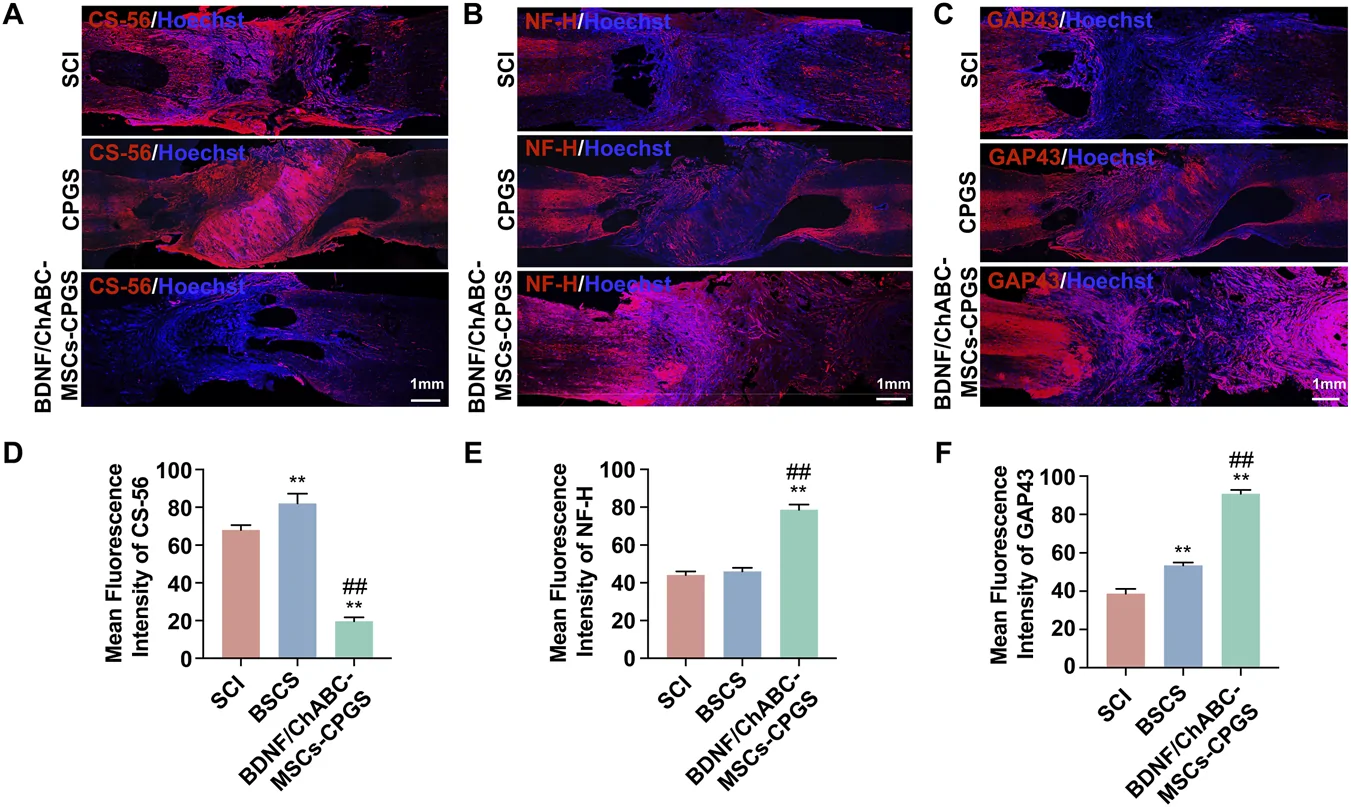
BDNF/ChABC-MSCs-CPGS modulates CSPG deposition and enhances axonal regeneration–associated markers after SCI. **(A)** Representative immunofluorescence images of CS-56 (CSPGs) determined via Hoechst nuclear counterstaining in SCI, CPGS, and BDNF/ChABC-MSCs-CPGS groups. **(B)** Representative immunofluorescence images of NF-H with Hoechst counterstaining in SCI, CPGS, and BDNF/ChABC-MSCs-CPGS groups. **(C)** Representative immunofluorescence images of GAP43 determined via Hoechst counterstaining in SCI, CPGS, and BDNF/ChABC-MSCs-CPGS groups. **(D)** Calculation of the mean fluorescence intensity of CS-56. **(E)** Quantification of mean fluorescence intensity of NF-H. **(F)** Quantification of mean fluorescence intensity of GAP43. Scale bar = 1 mm *P < 0.05, **P < 0.01 versus the SCI group; #*P* < 0.05, ##*P* < 0.01 versus the CPGS group.

Following BDNF/ChABC-MSCs-CPGS treatment, immunofluorescence analysis at the molecular and cellular levels revealed a shift in the lesion microenvironment from an inhibitory state to a more permissive, regenerative milieu. CS-56, a common marker for CSPG deposition, is typically increased with the accumulation of glial scar-associated inhibitory extracellular matrix (Tang et al., 2025). Compared to controls, the BDNF/ChABC-MSCs-CPGS group exhibited a marked reduction in CS-56 immunoreactivity (Figures 6A,D), indicating decreased intact CSPG-associated inhibitory matrix at the lesion site. However, because CS-56 exclusively marks intact chondroitin sulfate epitopes, this reduction indicates a less inhibitory microenvironment but cannot separate direct ChABC-mediated digestion from potential variations in endogenous matrix production induced by BDNF treatment. This suggests more favorable substrate conditions for axonal regrowth. In parallel, NF-H, a well-established marker of mature axonal structures (Zhou et al., 2023), was increased (Figures 6B,E), suggesting improved axonal preservation and structural continuity, while GAP43, a canonical regeneration-associated protein linked to axonal sprouting and elongation (Kisucká et al., 2026), was also elevated (Figures 6C,F). These findings collectively indicate that the CPGS may serve as a local cell-delivery matrix that supports local microenvironmental remodeling, thereby contributing to the subsequent neural axonal extension and functional recovery following SCI.

## 6 Discussion

Neurorepair after SCI is a complex and dynamic process involving remodeling of the injury microenvironment, intercellular signaling, immune regulation, and activation of endogenous regenerative mechanisms (Hu et al., 2023; Ma and Li, 2025; Gao et al., 2024). Accordingly, this study developed an integrated repair construct that combines therapeutic factor delivery, genetically engineered MSCs, and tissue-engineered structural guidance to provide complementary spatiotemporal modulation of the injured spinal cord microenvironment.

Regarding therapeutic factors, the influence of CSPGs on SCI is temporally dependent, offering protection during the acute phase but contributing to inhibition in later stages (Kisucká et al., 2026; Francos-Quijorna et al., 2022). Therefore, ChABC-based therapy should be administered in a time-controlled manner, whereas neurotrophic factors such as BDNF should be administered as early and continuously as possible (Huang et al., 2025). The Tet-On gene system enables precise temporal control of ChABC expression, while MSCs facilitate sustained, localized delivery of endogenous BDNF (Khanteymoori et al., 2025; Kim et al., 2021). In this study, ChABC expression was initiated 1 week post-SCI, degrading CSPGs to clear matrix barriers for neural regeneration. Precise temporal regulation prevents the negative consequences of early ChABC expression and optimizes the extracellular microenvironment for later cell activities.

At the stem cell level, combined BDNF and ChABC expression had a highly selective and targeted effect on the MSC transcriptome. Although the number of DEGs was small, these changes implicated several potentially relevant signaling pathways. Both transcriptomic analysis and protein validation showed that the dual gene modification activated key signaling pathways in MSCs, including FAK, MAPK, mTOR, and Hedgehog. Phosphorylation of FAK at Y397, a critical molecule for sensing extracellular matrix signals, was significantly increased in engineered MSCs. This enhanced the ability of BDNF/ChABC-MSCs to respond to the external environment and promoted the enrichment of genes involved in cell movement and directional migration (Gultian et al., 2022; Mohd Arizam et al., 2025; Hu et al., 2017). The activation of the MAPK pathway, particularly the specific phosphorylation of JNK and ERK1/2, further enhanced MSC survival and adaptability in complex microenvironments (Chen et al., 2024b; Fan et al., 2023). Activation of the mTOR pathway facilitated protein synthesis essential for cellular repair, thereby supporting cell proliferation and survival (Jhanwar-Uniyal et al., 2024; Vargova et al., 2021; Zhang et al., 2025). Upregulation of GLI1, the Hedgehog pathway’s core effector protein, played a key role in neurodevelopment and repair. Specifically, GLI1 regulated transcription factor expression, promoting neuronal differentiation and synapse reconstruction, which may participate in the early phase of microenvironmental remodeling. However, these observations represent repair-associated signaling alterations rather than definitive axonal outcomes, and further investigation is required to establish exact functional causality (Du et al., 2025; Z et al., 2024).

The activation of these four core signaling pathways likely involves multiple interactions. Based on established signaling models, FAK, a key upstream regulator of the PI3K/Akt axis, may indirectly modulate GLI1 protein stability and mTOR pathway activation via downstream kinase activity (Hu et al., 2024b; Sicurella et al., 2025). Although further inhibitor experiments are needed to elucidate the precise molecular mechanisms, the concurrent upregulation of p-FAK, p-mTOR, and GLI1 protein levels observed in this study suggests that MSCs, when stimulated with BDNF/ChABC, likely establish an integrated regulatory network. This network, mediated by FAK, integrates metabolic support with structural remodeling (Sicurella et al., 2025; Chen et al., 2020; Tu et al., 2025). By endogenously reprogramming multiple core signaling pathways, engineered MSCs exhibit enhanced biological function, establishing a signaling foundation that supports their role in SCI repair. While BDNF/ChABC-MSCs exhibit activation-associated changes in FAK, MAPK, mTOR, and Hedgehog signaling pathways, the current results do not formally demonstrate pathway synergy or direct causal interactions. Further mechanistic studies would be required to establish synergy.

At the tissue engineering level, the CPGS functions as a cell carrier, demonstrating exceptional biocompatibility and providing spatial guidance for neural repair via its unique physical morphology and physicochemical properties. The 19 longitudinal microchannels within the CPGS offer precise directional cues for axonal regeneration, effectively shortening the path for regenerated fibers to traverse the injury cavity. A critical design objective for the longitudinal guidance scaffold was the maintenance of a stable microchannel architecture to ensure predictable linear axonal orientation (Sousa et al., 2023). To explicitly differentiate our platform from existing channelized hydrogel scaffolds, the construct was engineered and benchmarked against the state of the art across three distinct axes: fabrication approach, channel geometry, and mechanical performance. Regarding the fabrication approach, rather than utilizing high-resolution but technically complex automated additive manufacturing setups that often introduce multi-layer structural heterogeneity (Yuan et al., 2022; Kim et al., 2024), we implemented an operationally streamlined thermal-assisted mold casting strategy using parallel stainless steel wire templates. Geometrically, cross-sectional analysis validates that CPGS contains a highly regularized 19-microchannel array featuring a constant luminal of Ø0.2 mm, which optimizes the spatial orientation for cellular alignment compared to randomized electrospun variants. Mechanically, our monolithic 10% Porous-GelMA matrix delivers a compressive modulus of ∼26.4 kPa, which strategically matches the mechanical upper boundary of host tissue substance, successfully resisting premature structural deformation while guaranteeing long-term luminal patency (Jin et al., 2023). The selected 10% Porous-GelMA CPGS should therefore be interpreted as an engineering compromise rather than a mechanically matched spinal cord substitute. While its stiffness supports surgical handling and microchannel patency, stiffness mismatch may influence host foreign body responses, MSC immunomodulatory behavior, and integrin–FAK-related mechanotransduction (Zheng et al., 2025). Because these biological consequences were not directly measured in the present study, they are acknowledged as potential mechanobiological limitations and important targets for future scaffold optimization. Consequently, the CPGS provides physical support to reduce cavity size and collagen deposition, while simultaneously guiding the directional regeneration of NF-H positive nerve fibers and synaptic regeneration mediated by GAP43 through the combined action of BDNF/ChABC-MSCs.

## 7 Conclusion

In conclusion, this study successfully constructed a channeled porous-GelMA scaffold (CPGS) loaded with genetically engineered MSCs capable of inducible ChABC and constitutive BDNF expression. In a rat complete spinal cord transection model, implantation of this integrated system significantly reduced the post-injury cavity, reduced scar-associated matrix deposition at the final endpoint, enhanced axonal preservation and regrowth, and supported the remodeling of the lesion microenvironment, ultimately contributing to the improved outcomes of hindlimb locomotor function.

## 8 Limitation

We explicitly acknowledge several major limitations in the current study that temper the immediate translational interpretation of our findings. First, the absence of full factorial animal control groups (e.g., MSCs alone, or single-factor engineered cells expressing only BDNF or ChABC) prevents the definitive dissection of the individual or interactive contributions of each therapeutic component to the observed functional recovery. Second, while the 10% Porous-GelMA scaffold successfully supported cell distribution *in vitro*, its static compressive properties do not fully represent the dynamic mechanical performance—such as tensile strength and cyclic fatigue resistance—required under physiological spine loading *in vivo*. Third, the selected 1-week post-injury delay for Dox induction and the 8-week terminal sampling point represent single static parameters derived from literature. Crucially, direct validation showing that systemic Dox successfully penetrates the blood-spinal cord barrier at effective therapeutic concentrations, that ChABC is efficiently secreted into the extracellular space *in vivo*, and that the enzyme retains optimal activity within the complex inflammatory milieu of SCI was not performed. Furthermore, without a standalone BDNF-MSCs-CPGS control group, the potential contribution of altered fibrotic signaling to the observed CS-56 reduction cannot be independently decoupled from ChABC-mediated digestion. Finally, the behavioral evaluation relied exclusively on gross locomotor BBB scoring; more granular assessments of fine motor coordination (e.g., grid walk test) or motor-evoked potential (MEP) electrophysiological recordings are required in future studies to rigorously confirm functional axonal reconnection. Additionally, although GLI1 upregulation may be associated with repair-related signaling, sustained Hedgehog/GLI1 activation should not be interpreted as uniformly beneficial. GLI1 is a terminal transcriptional effector of the Hedgehog pathway, and aberrant GLI1 activation has been linked to cancer-associated phenotypes, including enhanced proliferation, survival, stemness, angiogenesis, metastasis, metabolic reprogramming, and therapy resistance (Avery et al., 2021; Sigafoos et al., 2021). Therefore, although the present study observed GLI1 upregulation as a protein-level signaling-associated change in BDNF/ChABC-MSCs, we did not evaluate long-term proliferation kinetics, lineage fate, ectopic differentiation, or tumorigenicity of transplanted MSCs *in vivo*. These safety issues should be systematically assessed in long-term preclinical studies before translational application.

## Data Availability

The raw RNA-Seq data have been deposited in the NCBI BioProject database under accession number PRJNA1430096.

## References

[B1] Avery J. T. Zhang R. Boohaker R. J. (2021). GLI1: a therapeutic target for cancer. Front. Oncol. 11, 2021. 10.3389/fonc.2021.673154 PMC8186314 34113570

[B2] Basso D. M. Beattie M. S. Bresnahan J. C. (1995). A sensitive and reliable locomotor rating scale for open field testing in rats. J. Neurotrauma 12 (1), 1–21. 10.1089/neu.1995.12.1 7783230

[B3] Behtaj S. Ekberg J. A. K. St John J. A. (2022). Advances in electrospun nerve guidance conduits for engineering neural regeneration. Pharmaceutics 14 (2), 219. 10.3390/pharmaceutics14020219 35213952 PMC8876219

[B4] Chen J. Zhu Y. Zhao D. Zhang L. Zhang J. Xiao Y. (2020). Co-targeting FAK and Gli1 inhibits the tumor-associated macrophages-released CCL22-mediated esophageal squamous cell carcinoma malignancy. MedComm 4 (6), 10.1002/mco2.381 PMC10576977 37846367

[B5] Chen K. Yu W. Zheng G. Xu Z. Yang C. Wang Y. (2024a). Biomaterial-based regenerative therapeutic strategies for spinal cord injury. NPG Asia Mater. 16 (1), 5. 10.1038/s41427-023-00526-4

[B6] Chen Z. Xia X. Yao M. Yang Y. Ao X. Zhang Z. (2024b). The dual role of mesenchymal stem cells in apoptosis regulation. Cell. Death and Dis. 15 (4), 250. 10.1038/s41419-024-06620-x 38582754 PMC10998921

[B7] Cieri M. B. Ramos A. J. (2025). Astrocytes, reactive astrogliosis, and glial scar formation in traumatic brain injury. Neural Regen. Res. 20 (4), 973–989. 10.4103/nrr.nrr-d-23-02091 38989932 PMC11438322

[B8] Clifford T. Finkel Z. Rodriguez B. Joseph A. Cai L. (2023). Current advancements in spinal cord injury research-glial scar formation and neural regeneration. Cells 12 (6), 853. 10.3390/cells12060853 36980193 PMC10046908

[B9] Covache-Busuioc R.-A. Toader C. Rădoi M. P. Șerban M. (2025). Precision recovery after spinal cord injury: integrating CRISPR technologies, AI-Driven therapeutics, single-cell omics, and system neuroregeneration. Int. J. Mol. Sci. 26 (14), 6966. 10.3390/ijms26146966 40725213 PMC12295970

[B10] Das A. T. Tenenbaum L. Berkhout B. (2016). Tet-On systems for doxycycline-inducible gene expression. Curr. Gene Ther. 16 (3), 156–167. 10.2174/1566523216666160524144041 27216914 PMC5070417

[B11] Deznabi N. Hosseini S. Rajabi M. (2023). Neurotrophic factors-based therapeutic strategies in the spinal cord injury: an overview of recent preclinical studies in rodent models. Egypt. J. Neurology, Psychiatry Neurosurg. 59 (1), 63. 10.1186/s41983-023-00661-3

[B12] Du M. Ji X. Chen W. (2025). Sonic hedgehog signaling in spinal cord injury: mechanisms and therapeutic implications. Front. Mol. Neurosci. 18, 2025. 10.3389/fnmol.2025.1624501 PMC12344183 40808911

[B13] Fan M. Tong P. Yan L. Li T. Ren J. Huang J. (2023). Detrimental alteration of mesenchymal stem cells by an articular inflammatory microenvironment results in deterioration of osteoarthritis. BMC Med. 21 (1), 215. 10.1186/s12916-023-02923-6 37337188 PMC10280917

[B14] Fiford R. J. Bilston L. E. (2005). The mechanical properties of rat spinal cord *in vitro* . J. Biomech. 38 (7), 1509–1515. 10.1016/j.jbiomech.2004.07.009 15922762

[B15] Francos-Quijorna I. Sánchez-Petidier M. Burnside E. R. Badea S. R. Torres-Espin A. Marshall L. (2022). Chondroitin sulfate Proteoglycans prevent immune cell phenotypic conversion and inflammation resolution via TLR4 in rodent models of spinal cord injury. Nat. Commun. 13 (1), 2933. 10.1038/s41467-022-30467-5 35614038 PMC9133109

[B16] Gao Y. Wang Y. Wu Y. Liu S. (2024). Biomaterials targeting the microenvironment for spinal cord injury repair: progression and perspectives. Front. Cell. Neurosci. 18, 2024. 10.3389/fncel.2024.1362494 PMC11111957 38784712

[B17] Gultian K. A. Gandhi R. Sarin K. Sladkova-Faure M. Zimmer M. de Peppo G. M. (2022). Human induced mesenchymal stem cells display increased sensitivity to matrix stiffness. Sci. Rep. 12 (1), 8483. 10.1038/s41598-022-12143-2 35589731 PMC9119934

[B18] Haratizadeh S. Liu H. Li H. Adeli M. All A. H. (2025). Biomaterials and cell-based therapy post spinal cord injury. J. Transl. Med. 23 (1), 1042. 10.1186/s12967-025-06974-6 41039542 PMC12490130

[B19] Hu Y. Lu J. Xu X. Lyu J. Zhang H. (2017). Regulation of focal adhesion turnover in SDF-1α-stimulated migration of mesenchymal stem cells in neural differentiation. Sci. Rep. 7 (1), 10013. 10.1038/s41598-017-09736-7 28855566 PMC5577153

[B20] Hu X. Xu W. Ren Y. Wang Z. He X. Huang R. (2023). Spinal cord injury: molecular mechanisms and therapeutic interventions. Signal Transduct. Target. Ther. 8 (1), 245. 10.1038/s41392-023-01477-6 37357239 PMC10291001

[B21] Hu Y. Chen H. Yang M. Xu J. Liu J. He Q. (2024a). Hepatocyte growth factor facilitates the repair of spinal cord injuries by driving the chemotactic migration of mesenchymal stem cells through the β-catenin/TCF4/Nedd9 signaling pathway. Stem Cells 42 (11), 957–975. 10.1093/stmcls/sxae055 39269318

[B22] Hu H.-H. Wang S. Q. Shang H. L. Lv H. F. Chen B. B. Gao S. G. (2024b). Roles and inhibitors of FAK in cancer: current advances and future directions. Front. Pharmacol. 15, 2024. 10.3389/fphar.2024.1274209 PMC10895298 38410129

[B23] Huang Z. Li J. Wo J. Li C. Wu Z. Deng X. (2025). Intranasal delivery of brain-derived neurotrophic factor (BDNF)-loaded small extracellular vesicles for treating acute spinal cord injury in rats and monkeys. J. Extracell. Vesicles 14 (4), e70066. 10.1002/jev2.70066 40194993 PMC11975507

[B24] Jhanwar-Uniyal M. Zeller S. L. Spirollari E. Das M. Hanft S. J. Gandhi C. D. (2024). Discrete mechanistic target of rapamycin signaling pathways, stem cells, and therapeutic targets. Cells 13 (5), 409. 10.3390/cells13050409 38474373 PMC10930964

[B25] Jin C. Zhu R. Wang Z. w. Li Y. Ni H. f. Xu M. l. (2023). Dynamic changes in mechanical properties of the adult rat spinal cord after injury. Acta Biomater. 155, 436–448. 10.1016/j.actbio.2022.11.041 36435440

[B26] Kalimuthu S. Oh J. M. Gangadaran P. Zhu L. Lee H. W. Jeon Y. H. (2017). Genetically engineered suicide gene in mesenchymal stem cells using a Tet-On system for anaplastic thyroid cancer. PLoS One 12 (7), e0181318. 10.1371/journal.pone.0181318 28727740 PMC5519161

[B27] Khanteymoori A. Peterson C. Atamny R. Hohenhaus M. Beck J. Howells D. W. (2025). Targeting nerve fiber outgrowth inhibition after experimental spinal cord injury: a systematic review and meta-analysis of chondroitinase ABC. Neurorehabil Neural Repair 39 (4), 312–320. 10.1177/15459683241311337 39772811

[B28] Kim H. J. Bayarsaikhan D. Lee J. Bayarsaikhan G. Lee B. (2021). Brain-derived neurotrophic factor secreting human mesenchymal stem cells improve outcomes in Rett syndrome mouse models. Front. Neurosci. 15, 725398. 10.3389/fnins.2021.725398 34690674 PMC8526791

[B29] Kim D. Y. Liu Y. Kim G. An S. B. Han I. (2024). Innovative strategies in 3D bioprinting for spinal cord injury repair. Int. J. Mol. Sci. 25 (17), 9592. 10.3390/ijms25179592 39273538 PMC11395085

[B30] Kisucká A. Bimbová K. Kuruc T. Ileninová M. Kuchárová K. Ihnátová L. (2026). Molecular changes in CSPG and glial scar markers in response to subpial chondroitinase ABC treatment following spinal cord injury. Eur. J. Neurosci. 63 (1), e70389. 10.1111/ejn.70389 41518155

[B31] Liu J. Ji Z. He Q. Chen H. Xu X. Mei Q. (2024). Direct conversion of human umbilical cord mesenchymal stem cells into dopaminergic neurons for parkinson's disease treatment. Neurobiol. Dis. 201, 106683. 10.1016/j.nbd.2024.106683 39343249

[B32] Ma W. Li X. (2025). Spinal cord injury repair based on drug and cell delivery: from remodeling microenvironment to relay connection formation. Mater. Today Bio 31, 101556. 10.1016/j.mtbio.2025.101556 40026622 PMC11871491

[B33] Mohd Arizam D. N. Nordin F. Ahmad A. Ahmad Amin Noordin K. B. (2025). Integrin signaling pathways in mesenchymal stem cells. Stem Cell. Res. and Ther. 16 (1), 472. 10.1186/s13287-025-04608-8 40877985 PMC12395929

[B34] Sharifi A. Zandieh A. Behroozi Z. Hamblin M. R. Mayahi S. Yousefifard M. (2022). Sustained delivery of chABC improves functional recovery after a spine injury. BMC Neurosci. 23 (1), 60. 10.1186/s12868-022-00734-8 36307768 PMC9615228

[B35] Sicurella M. De Chiara M. Neri L. M. (2025). Hedgehog and PI3K/Akt/mTOR signaling pathways involvement in leukemic malignancies: crosstalk and role in cell death. Cells 14 (4), 269. 10.3390/cells14040269 39996741 PMC11853774

[B36] Sigafoos A. N. Paradise B. D. Fernandez-Zapico M. E. (2021). Hedgehog/GLI signaling pathway: transduction, regulation, and implications for disease. Cancers (Basel) 13 (14), 3410. 10.3390/cancers13143410 34298625 PMC8304605

[B37] Sousa J. P. M. Stratakis E. Mano J. Marques P. A. (2023). Anisotropic 3D scaffolds for spinal cord guided repair: current concepts. Biomater. Adv. 148, 213353. 10.1016/j.bioadv.2023.213353 36848743

[B38] Takiguchi M. Miyashita K. Yamazaki K. Funakoshi K. (2022). Chondroitinase ABC administration facilitates serotonergic innervation of motoneurons in rats with complete spinal cord transection. Front. Integr. Neurosci. 16, 2022. 10.3389/fnint.2022.881632 35845919 PMC9280451

[B39] Tang W. Wang A. Liu S. Wen G. Qi H. Gu Y. (2025). Calycosin regulates astrocyte reactivity and astrogliosis after spinal cord injury by targeting STAT3 phosphorylation. J. Neuroimmunol. 400, 578535. 10.1016/j.jneuroim.2025.578535 39954615

[B40] Trigo C. M. Rodrigues J. S. Camões S. P. Solá S. Miranda J. P. (2025). Mesenchymal stem cell secretome for regenerative medicine: where do we stand? J. Adv. Res. 70, 103–124. 10.1016/j.jare.2024.05.004 38729561 PMC11976416

[B41] Tu Y. Pan B. Wu X. Hu X. Li Q. Zhang Z. (2025). Focal adhesion kinase acts on the PI3K/Akt/mTOR pathway to modulate wear particle-induced autophagy in macrophages. Int. Immunopharmacol. 163, 115246. 10.1016/j.intimp.2025.115246 40706210

[B42] Vargova I. Machova Urdzikova L. Karova K. Smejkalova B. Sursal T. Cimermanova V. (2021). Involvement of mTOR pathways in recovery from spinal cord injury by modulation of autophagy and immune response. Biomedicines 9 (6), 593. 10.3390/biomedicines9060593 34073791 PMC8225190

[B43] Varkouhi A. K. Monteiro A. P. T. Tsoporis J. N. Mei S. H. J. Stewart D. J. Dos Santos C. C. (2020). Genetically modified mesenchymal stromal/stem cells: application in critical illness. Stem Cell. Rev. Rep. 16 (5), 812–827. 10.1007/s12015-020-10000-1 32671645 PMC7363458

[B44] Wu Y. Y. Gao Y. M. Feng T. Rao J. S. Zhao C. (2025). Enhancing functional recovery after spinal cord injury through neuroplasticity: a comprehensive review. Int. J. Mol. Sci. 26 (14), 6596. 10.3390/ijms26146596 40724846 PMC12294513

[B45] Xia Y. Zhu J. Yang R. Wang H. Li Y. Fu C. (2023). Mesenchymal stem cells in the treatment of spinal cord injury: mechanisms, current advances and future challenges. Front. Immunol. 14, 1141601. 10.3389/fimmu.2023.1141601 36911700 PMC9999104

[B46] Xu J.-H. Xu S. Q. Ding S. L. Yang H. Huang X. Shi H. F. (2022). Bone marrow mesenchymal stem cells alleviate the formation of pathological scars in rats. Regen. Ther. 20, 86–94. 10.1016/j.reth.2022.03.004 35509267 PMC9048073

[B47] Yuan T. Y. Zhang J. Yu T. Wu J. P. Liu Q. Y. (2022). 3D bioprinting for spinal cord injury repair. Front. Bioeng. Biotechnol. 10, 847344. 10.3389/fbioe.2022.847344 35519617 PMC9065470

[B48] Yusop N. Battersby P. Alraies A. Sloan A. J. Moseley R. Waddington R. J. (2018). Isolation and characterisation of mesenchymal stem cells from rat bone marrow and the endosteal niche: a comparative study. Stem Cells Int. 2018, 6869128. 10.1155/2018/6869128 29765418 PMC5885338

[B49] Zhang L. Wang J. Xu N. Guo J. Lin Y. Zhang X. (2024). POU3F4 up-regulates Gli1 expression and promotes neuronal differentiation and synaptic development of hippocampal neural stem cells. Stem Cell. Res. and Ther. 15 (1), 440. 10.1186/s13287-024-04043-1 39563384 PMC11577835

[B50] Zhang H. Xiao X. Pan Z. Dokudovskaya S. (2025). mTOR signaling networks: mechanistic insights and translational frontiers in disease therapeutics. Signal Transduct. Target. Ther. 10 (1), 428. 10.1038/s41392-025-02493-4 41469379 PMC12753737

[B51] Zhang J. Xiang L. Cai X. Geng Y. Wu Y. Shi J. (2026). 3D-MSCs apoptotic bodies-integrated conductive hydrogel mitigates spinal cord injury via immunoregulation and alleviating neuronal pyroptosis. Bioact. Mater. 60, 704–723. 10.1016/j.bioactmat.2026.01.043 41704618 PMC12907102

[B52] Zheng Y. Nützl M. Schackel T. Chen J. Weidner N. Müller R. (2025). Biomaterial scaffold stiffness influences the foreign body reaction, tissue stiffness, angiogenesis and neuroregeneration in spinal cord injury. Bioact. Mater. 46, 134–149. 10.1016/j.bioactmat.2024.12.006 39760066 PMC11700269

[B53] Zhou L. Lin D. Xu G. Wang X. Chen Z. Wang D. (2023). Alteration of neurofilament heavy chain and its phosphoforms reveals early subcellular damage beyond the optic nerve head in glaucoma. Front. Neurology 14, 2023. 10.3389/fneur.2023.1091697 PMC10073422 37034083

