# Supplementary material for: Combined treatment of spinal cord injury using channeled Porous-GelMA scaffold loaded with genetically engineered MSCs expressing inducible ChABC and constitutive BDNF
Source: Front Bioeng Biotechnol. 2026 Aug 10;14:1838918. doi: 10.3389/fbioe.2026.1838918 (PMC13500550; doi:10.3389/fbioe.2026.1838918)
Supplement: Supplementary file 1 [file Supplementaryfile1.docx]

**Supplementary Materials**

Graphical Abstract Legend:

This study developed a combined therapeutic system to repair spinal cord injuries (SCI) by integrating genetically engineered MSCs into channeled porous-GelMA scaffolds (CPGS). The MSCs were programmed via lentiviral transduction to secrete ChABC under the control of Doxycycline induction and to constitutively express BDNF. After transplantation into rats with acute SCI, this CPGS-based system effectively remodeled the inhibitory microenvironment by reducing CSPG-rich scars and lesion cavities, which promoted axon regeneration and significant functional recovery.

Supplementary Figure 1:

All mesenchymal stem cell (MSC) groups were genetically modified using the Tet3G regulatory system and a green fluorescent protein (GFP) reporter gene. The GFP reporter, present in all experimental conditions, enabled cell tracking and downstream validation. Specifically, the Control Group MSCs were transduced with the Tet3G element and GFP alone, serving as a baseline for comparison. The BDNF Group cells were engineered to constitutively secrete BDNF and express GFP. In the ChABC Group, MSCs featured a Doxycycline-inducible ChABC-Flag gene, with GFP co-expressed as a reporter of successful transduction and gene activation. Finally, the BDNF/ChABC Group combined these elements, enabling simultaneous constitutive BDNF secretion, inducible ChABC-Flag expression, and robust GFP fluorescence for longitudinal monitoring of the transplanted cells within the spinal cord microenvironment.

Supplementary Figure 2

Supplementary method: Dimethylmethylene blue (DMMB) assay for indirect quantification of chondroitinase ABC (ChABC) activity. The enzymatic activity and secretory kinetics of active ChABC in culture supernatants were determined via an extracellular chondroitin sulfate proteoglycan (CSPG) degradation-based 1,9-dimethylmethylene blue (DMMB) colorimetric assay. Briefly, the reaction system was configured in a 96-well plate with a total volume of 20 μL per well. Each well contained bovine nasal cartilage-derived CSPG (0.25 mg/mL; Sigma-Aldrich, C3329) incubated at 37°C for 15 min with either culture supernatants from the engineered MSC groups or serial dilutions of active reference ChABC (0.1, 0.05, 0.025, 0.0125, 0.00625, and 0.003125 U) to optimize complete digestion kinetics. For baseline controls, CSPG incubated with 0.1% bovine serum albumin (BSA) served as the no-enzyme control, while ddH2O incubated with ChABC (0.003125 U) served as the substrate-free blank. Following the 15-min incubation, 160 μL of DMMB colorimetric reagent (Sigma-Aldrich, 341088) was introduced into each well and mixed gently. The absorbance values were captured immediately at a wavelength of 595 nm utilizing a microplate reader. A standard calibration curve was generated based on the residual GAG content against active ChABC concentrations, and the functional enzyme activity in the harvested culture supernatants was quantified accordingly based on the relative reduction in GAG levels compared with control reactions.

| 1 | 2 | 3 | 4 | 5 | 6 | 7 | 8 |
| --- | --- | --- | --- | --- | --- | --- | --- |
| CSPG 0.25mg/ml | | | | | | | ddH_2_0 |
| 0.1% BSA | ChABC 0.1 U | ChABC 0.05 U | ChABC 0.025 U | ChABC 0.0125 U | ChABC 0.00625 U | ChABC 0.003125 U | ChABC 0.003125 U |

The enzymatic activity and relative amount of ChABC in the culture supernatants were determined based on the decrease in GAG levels compared with control reactions.

Figure legends: In vitro validation of ChABC secretory activity and functional kinetics. (A) Standard calibration curve of active ChABC-mediated GAG cleaving kinetics (Y = 5.019*X + 0.1199). (B) Quantitative evaluation of active GAG release (U) in culture supernatants harvested from engineered ChABC-MSCs and combined BDNF/ChABC-MSCs under Dox induction, demonstrating robust extracellular secretory function with no statistically significant variance between the single and dual-transduced groups (n.s., P > 0.05).
